# Knowledge, attitude and practice for cervical cancer prevention and control among women of childbearing age in Hossana Town, Hadiya zone, Southern Ethiopia: Community-based cross-sectional study

**DOI:** 10.1371/journal.pone.0181415

**Published:** 2017-07-25

**Authors:** Yitagesu Habtu Aweke, Samuel Yohannes Ayanto, Tariku Laelago Ersado

**Affiliations:** 1 Department of Health Informatics, Hossana College of Health Sciences, Hossana, Ethiopia; 2 Department of Midwifery, Hossana College of Health Sciences, Hossana, Ethiopia; 3 Department of Clinical Nursing, Hossana College of Health Sciences, Hossana, Ethiopia; Duke University, UNITED STATES

## Abstract

**Background:**

Cervical cancer is the second most common female cancer which Ethiopia put a strategic goal to reduce its incidence and mortality by 2020. Lack of knowledge and poor attitude towards the disease and risk factors can affect screening practice and development of preventive behavior for cervical cancer. The aim of this study was to assess knowledge, attitude, practices and factors for each domain for cervical cancer among women of child bearing age in Hossana town, Southern, Ethiopia.

**Methods:**

Community based cross sectional study was carried out in June 2015. A total of 583 participants were selected using systematic random sampling technique. Pretested structured interviewer administered questionnaire was used to gather the data. Data were entered in to Epi Info software version 3.5.4 and exported to SPSS version 16 for descriptive and logistic regression analysis.

**Results:**

Two hundred seventy (46.3%) of the respondents had poor comprehensive knowledge. Only 58 (9.9%) of participants had been screed for the cervical cancer before the survey. Two hundred three (34.8%) of participants had negative attitude towards selected proxy variables. Not having health seeking behavior for cervical cancer [AOR: 5.45, 95% CI: (1.18, 30.58), P <0.031], had not ever received information about cervical cancer and its prevention [AOR: 2.63, 95%CI: (1.78,8.84), P < 0.018] and not actively seeking health information about cervical cancer [AOR: 6.25, (95%CI: (1.26, 31.06) P < 0.025] were significantly associated factors with poor knowledge. Poor knowledge score was associated with poor attitude [AOR: 56.51, 95%CI: (23.76, 134.37), P <0.001]. Had not ever received information about the disease from any source [AOR: 45.24, (95%CI: (11.47, 178.54), P <0.001] was significantly associated factor with not to be screened for the disease.

**Conclusion:**

This study highlighted the importance of awareness creation, increasing knowledge, promoting active searching for health information and experiences of receiving information from any information sources regarding cervical cancer. Therefore, it will be essential to integrate cervical cancer prevention strategies with other reproductive health services at all level of health care delivery system.

## Background

Cancer is increasingly growing as a major public health problem in both developed and developing countries amongst the chronic diseases [1]. Cancer can impose health, heavy economic and social burden. It is a global pandemic affecting both developed and developing regions, but it is rapidly increasing in low and middle-income countries, where resources for prevention, diagnosis and treatment are limited or non-existent [2].

Breast and cervical cancers are the leading cancers among women in developing countries, with estimated annual new cases of 882,900 and 444,500 respectively. More than 324,300 and 230,400 women die from these cancers every year, respectively. Globally, the number of new cases and deaths from cervical cancer is projected to continue to rise 720,415 and 394,905 respectively in 2025. A marked increase has been predicted specifically in GAVI-eligible countries, with a 58% expected increase in the number of new cases and a 63% expected increase in the number of related deaths [3].

In sub-Saharan Africa, 34.8 new cases of cervical cancer are diagnosed per 100,000 women annually, and 22.5 per 100,000 women die from the disease. This figure is higher when compared with 6.6 and 2.5 per 100 000 women, respectively, in North America. The marked differences can be explained by low preventive health behavior, lack of access to effective screening services that facilitate early detection and treatment [4].

Non-communicable diseases in general and cervical cancer in particular are becoming a double burden for Ethiopia. Although national representative survey is not currently available in Ethiopia, the International Agency for Research on Cancer (IARC) estimated that cancer is the second most cause of death and morbidity annually in 2012. Of which, cervical cancer is the second cause of death and morbidity next to breast cancer [2]. According to data from Addis Ababa population based cancer registry, cervical cancer was the second leading cancer comprising 10.8% of all cases of cancer [5].

Cervical cancer is mainly caused by sexually transmitted Human Papilloma Virus (HPV), which is the most common viral infection of the female reproductive tract. Almost all sexually active individuals will be infected with HPV at some point in their lives and some may be repeatedly infected [6]. Cervical cancer is one of the most easily preventable forms of female cancers. Yet, lack of knowledge about the disease and risk factors, beliefs about the disease, poor access to preventive services, affordability of the service and current health service system can affect decision to be screened for cervical cancer [6, 7, 8].

Despite the fact that multiple health needs compete for attention and limited funding resulting in reduced priority for implementing comprehensive cervical cancer prevention packages, the government of Ethiopia and supporting organizations have been starting to work on cervicalcancer prevention and control activities. In line with this, a single-visit approach (SVA) is a simple and affordable screening procedure that is being implemented recently [9]. Furthermore, increasing awareness, knowledge, attitude and practices of the community are strategic interventions expected to be a tool for primary prevention, early detection, diagnosis and treatment and palliative care services according to health sector transformation plan of Ethiopia [10].

Ethiopia has put in place a strategic goal to reduce cancer incidence and mortality by 15% by 2020. Because its the burden and high mortality, cervical cancer is considered priority cancer for intervention. This ambitious plan aimed to reach 50% of the population with prevention awareness information, 80% coverage of each year’s target cohort of girls aged 9 to 13 with vaccination against HPV, reduce the exposure to HPV infections, 80% coverage of Visual Inspection with Acetic Acid (VIA) to detect precancerous cervical lesions among non-symptomatic women aged 30–49 and increase awareness to 50% among the general population and health care providers of early signs and symptoms and opportunities for early detection of cervical cancer [11].

Despite cervical cancer control guidelines available, lack of communication strategy oncancer, lack of awareness of risk factors, lack of coordinated prevention activitiesand ownership, lack of Information Education and Communication /Behaviour Change Communication (IEC/BCC) materials on cancer in general and cervical cancer in particular,and lack of immunization against HPV are some of the challenges faced in Ethiopia. The current strategy on cancer in general and cervical cancer in particular focuses on prevention and control of smoking and other modifiable risk factors and enhancing health promotion, education and advocacy to improve public understanding [11].

Knowledge, attitude and practice level of the community is very essential about the signs and symptoms of cervical cancer, risk factors, benefits of early diagnosis and treatment, availability of health services and prevention methods (HPV vaccination) [12].The women’s knowledge and attitude about the disease is influenced by socio demographic factors and the availability and accessibility of health services. In turn, screening behavior is a complex outcome of many factors operating at individual, family, and community levels [13].

In Ethiopia, there is limited information regarding knowledge, attitude and practices and associated factors for cervical cancer prevention and control at community level as mentioned by the national strategic plan on prevention and control of non-communicable diseases to design and implement any intervention [5]. To the best of our knowledge, there are no community based studies conducted so far on target populations except few facility based studies on cervical cancer screening. Therefore, the objective of this study was to determine level of knowledge, attitude, practice and factors affecting these behavioral elements for prevention and control of cervical cancer in Hossana town Ethiopia.

## Methods

### Study area and period

This study was conducted in Hossana town, the capital of Hadiya Zone, SNNPR of Ethiopia in June 2015. The town is situated 232 Kms southwest of Addis Ababa and 194 kms northwest of the regional capital Hawassa. The town has one zonal hospital, three public health centres and more than 25 clinics of all types providing curative and preventive health serivices.The estimated population of the town was 101,849 in 2014. The estimated total households in the town was 20,785 and women of child bearing age (15–49) was 23,731.

It is reported that there have not been targeted interventions regarding non-communicable diseases prevention and control including cervical cancer by the zonal health department and other stakeholders of the health care system before this study. Health care facilities especially hospitals have a health education and information dissemination program for major communicable and non-communicable diseases including other reproductive health issues but have never covered issues regarding cervical cancer prevention, control and treatment.

### Study design

Community based cross-sectional study design was employed.

### Study participants

The source population was all child bearing women whose age ranged from 18 to 49 years. The study population was WCBA (18–49 years) who had the chance of being randomly selected from the source population at a household level. We excluded women who are not permanent residents of the town (less than six year), those who had any serious illness during data collection and who are less than 18 years from the study.

### Sample size

The sample size for the study was estimated by using single population proportion formula at 95% confidence level (CI), Z (1-ά/2) = 1.96), an expected poor knowledge proportion of 50% and, 5% margin of error. As to the researchers' knowledge, there is no study previously determined the proportion of the poor knowledge regarding cervical cancer near by the study area and the variation of the study population would be expected to be high which we need maximum sample size to detect the difference. Therefore, the proportion of 50% was considered to determine the minimum sample size required for the study. On the other hand, we used at least two stages down in the sampling process to reach to the final sampling unit. As a result, we used a design effect of 1.5 to multiply our sample size to minimize the variability and detect the effect observed regarding knowledge about cervical cancer and its prevention. Using the above assumptions, the sample size was calculated as follows.

$$n=\frac{{Z\frac{\alpha }{2}}^{2}\;P(1-P)}{d^{2}}$$

$$n=\frac{{\left(1.96\right)}^{2}\;0.5(1-0.5)}{{\left(0.05\right)}^{2}}=384$$

We considered the none-response rate of 5% in the estimation of the sample size required for the study. Therefore, the final sample size was **595** women of child bearing age in the age group of 18–49 years.

### Sampling techniques

Firstly, all the kebeles found in the three sub cities were listed in a frame. Then, a total of five Kebeles were randomly selected using lottery method. Again list of women of the reproductive age group in each kebele were extracted from the town’s urban health extension unit as a sampling frame. The size of households consisting eligible population to be selected from each kebele was determined proportionally based on the size of the study units and k^th^ value was computed for each selected kebele. Spinning pen method was applied at the center of each kebele to decide the direction and start of the data collection process. We used systematic random sampling technique to select the study units. Lottery method was used to select one woman interviewee whenever two or more women of child bearing age (18–49 years) exist in the selected household. The data collection process was continued until the sample size for that particular kebele has been saturated.

### Data collection and measurement

Pretested structured questionnaire was used to collect data from each study subject. The questionnaire was adapted from related literatures [14–15] with slight modification in line with the objectives of this particular study and to fit to the local context. The questionnaire was first prepared in English, translated into Amharic and then back translated to English to check for its consistency. The questionnaire was pretested in 5% of the study population in kebeles having similar context with our study setting but not selected as part of the our study setting. Data collection was conducted through face to face interview by ten female diploma holders in health fields. The questionnaire was completed after obtaining verbal consent from the participants. The completed questionnaires were collected on a daily bases to check for its consistency and completeness. Repeated visits were made in a case when the interviewees were unavailable in their homes during the time of visit

Data collectors and supervisors were trained for two days on the procedures of data collection community entry and communication skills. Day to day supervision was carried out for the entire length of the data collection period by two trained supervisors. Questionnaires with incomplete information were presented back to the respective data collector to complete it by making repeated revisits.

Data were collected on knowledge,attitude twards and practice to cervical cancer and its socio-demographic factors and behavioral factors. In addition data were collected on health service factors including availability of the service, access to the service, availability of information education and communication and active searching of health information regarding cervical cancer prevention.

### Study variables

**Dependent variables** were knowledge of respondents, attitude towards cervical cancer and screening practice defined in operational definition section.

**Independent variables** were socio-demographic, behavioral and health service related factors for cervical cancer prevention and control.

### Operational definitions

**Knowledge:** We used a twenty nine items composite score of the knowledge to measure the knowledge level of respondents regarding vulnerable groups, risk factors, signs and symptoms and prevention methods of cervical cancer. The cumulative mean score of knowledge of participants about cervical cancer was estimated using mean score. Based on this, those who had scored less than the mean were considered to have **poor knowledge** and those who had scored greater than or equal to the mean value were considered as having **good knowledge**.

**Attitude**: We selected seven items to measure attitude towards proxy variables of perceived susceptibility, severity, benefit and barriers of screening and prevention of cervical cancer. We used five Likert scales (Strongly disagree, Disagree, Neutral, Agree and Strongly agree) to measure the level of agreement on each selected item. Finally, frequency of respondents who exclusively reported one of the three categories i.e. neutral, disagree or strongly disagree together was considered as having negative attitude while frequency of respondents who reported one of the two categories i.e. agree or strongly agree together was considered as having positive attitude.

**Practice:** Cervical cancer screening practice was assessed using questions having “Yes” or “No” response.

### Data processing and analysis

The collected data was cleaned and checked for consistency and completeness and entered into EPI info version 3.5.4 and exported to SPSS version 20 for descriptive and logistic regression analysis. Descriptive data analysis was used to describe the knowledge, attitude and practice factors for cervical cancer prevention and control. Different frequency tables, graphs and descriptive summaries were used to describe the variables. We used reverse coding using recode command of SPSS for attitude scales on selected statements so that agreements on seven statements went on similar direction i.e negative attitude or positive attitude towards proxy variables of interest. Multivariate logistic regression was used to identify associated factors with our three outcome variables (knowledge, attitude and practice) regarding cervical cancer, its risk factor and prevention. Odds ratio at 95% CI was computed to show the strength of the association between the outcome and the explanatory variables. All variables which showed statistically significant results with knowledge, attitude and practice for cervical cancer in bivariate logistic regression were entered to multivariate logistic regression to identify the independent contribution of each explanatory variable. P-value <0.05 was considered to decide statistically significant association between the independent and dependent variables.

### Ethical consideration

The institutional review board of the college reviewed and approved the research protocol before the research has began as per the set standard. Finally, the ethical clearance was obtained from institutional review board of Hossana College of Health Sciences. Official letter of permission was also obtained from respective administrative officials at each hierarchy in the study area. Information about the objectives of the study, confidentiality issues and the respondent’s autonomy was explained for each participant just before the commencement of data collection. Consent was obtained from each study participant to ensure voluntary participation and get the required information. We documented the participant consent with the recorded data is for atleast two years according to the ethical guidelines of the college’s institutional review board.

## Results

### Socio-demographic characteristics

The total size of the study subjects who were actual respondents during the data collection period was 583. Therefore, the response rate of the study was calculated to be 98%. The participants’ age ranged from 18 to 48. The median age of the study subjects was found to be 28 years with standard deviation of ±6.83. Most of the respondents, 366 (62.8%), were currently married. Among the total respondents, 388 (66.6%) were Protestant Christians, 123 (21.1%) were Orthodox Christians and Islam, Adventist, Catholic, Jehovah and Apostolic altogether constituted 12.3%. The three major ethnic groups among the study subjects were Hadiya 368 (63.1%), Kembata 79 (13.6%) and Amhara 57 (9.8%). Majority of the respondents, 127 (21.8%), were government employees in their occupation (Table 1).

**Table 1:** Socio-demographic characteristics of respondents of health seeking behavior in Hossana town Hadiya Zone, Ethiopia, June 2015.

Back ground variable	Categories	Frequency	%
Current Marital status (n = 583)	Married	366	62.8
	Single	149	25.6
	Widowed	26	4.5
	Separated	25	4.3
	Divorced	17	2.9
Religion (n = 583)	Protestant	388	66.6
	Orthodox	123	21.1
	Islam	33	5.7
	Others	39	6.6
Ethnicity (n = 583)	Hadiya	368	63.1
	Kembata	79	13.6
	Amhara	57	9.8
	Gurage	41	7.0
	Others*	38	6.6
Respondents’ age (n = 583)	< = 23	135	23.2
	24–27	128	22
	28–34	170	29.2
	> = 35	150	25.6
Respondents’ education (n = 583)	No education	74	12.7
	Primary education (1–8)	172	29.5
	Secondary education (9–12)	177	30.4
	Tertiary education (12 plus)	160	27.4
Respondents occupation (n = 583)	House wife	248	42.5
	Employee	127	21.8
	Student	85	14.6
	Merchant	71	12.2
	Others **	52	8.9
Parity (n = 583)	0	162	27.8
	1	82	14.1
	2–4	238	40.8
	> = 5	101	17.3
Husband's occupation (n = 389)	Employee	155	26.6
	Merchant	140	24
	Daily worker	30	5.1
	Farmer	21	3.6
	Others **	43	7.3
Husband's education (n = 389)	No education	14	2.4
	Primary education (1–8)	98	16.8
	Secondary education (9–12)	122	20.9
	Tertiary education (12 plus)	155	26.6
Monthly income in USD(n = 583)	<72	134	23.0
	72–143	212	36.4
	144–215	97	16.6
	>215	140	24.0

Others*Silte, Wolayita, Gamo

**daily labourer, house maid, farmers cattle feeders, coffee makers, etc

The minimum and maximum monthly income of study participants was 11USD and 1913 USD respectively. The average monthly income of the respondents’ family was 179.38 USD with the standard deviation of ±209.10 USD. The majority, 212 (36.4%) of the respondents’ families have their monthly income between 71.43–142.86 USD. Nearly quarter, 140 (24%) and 134 (23%) of the respondents’ families monthly income lies above the third quartile and below the first quartile values respectively. Majority 238 (40.8%) of the respondents were multi-paras followed by nulliparous which accounted 162 (27.8%) of the study participants. The average parity of the study participants was approximately 2.3 with the standard deviation of ±2.2 (Table 1).

### Knowledge of women on cervical cancer

According to operational definition given in methods section, 270 (46.3%) of the respondents had poor knowledge i.e. scored less than the mean (7.57 ± SD 6.61). Whereas, 313 (53.7%) of respondents had good knowledge i.e. scored greater than or equal to the mean.

Less than half, 254 (43.6%) of the respondents believed that all women are at risk of getting cervical cancer while 216 (37.0%) of them did not know which women are at risk of getting the disease. Two hundred twenty three (38.3%) of participants had no idea what factors raise chance of getting cervical cancer whereas 165 (28.3%) of participants reported that having multiple sexual partners is a risk factor for the disease. Similarly, more than a quarter, 209 (35.8%) of participants affirmed that they had no information about symptoms of cervical cancer while 220 (37.7%) of them indicated that persistent pelvic pain is the symptom of the disease (Table 2).

**Table 2:** Knowledge of respondents about risk groups, risk factors, signs and symptoms and methods of prevention of cervical cancer in Hossana town, Hadiya Zone, June 2015.

Risk group cited by respondents for pap smear*	**Frequency**	**Percent**
All women of child bearing age	254	43.6
Women with gynecological problems only	53	9.1
Pregnant women only	32	5.5
Sexually active women only	28	4.8
Do not know	216	37.0
Risk factors mentioned by respondents*		
Multiple sexual partners	165	28.3
Having many children	135	23.2
Starting to have sex before age 17	133	22.8
Having a weakened immunity	113	19.4
Having history of STI	80	13.7
Use of oral contraceptive pills	69	11.8
Smoking cigarette	55	9.4
Infection with human papilloma virus	52	8.9
Not using condom during sex	40	6.9
Family history of cervical cancer	19	3.3
I do not know	223	38.3
Signs and symptoms mentioned by respondents*		
Persistent pelvic pain	220	37.7
Abnormal vaginal bleeding	159	27.3
Abnormal vaginal discharge	118	20.2
Pain during sex	112	19.2
Other signs and symptoms	108	18.5
I don’t know	209	35.8
**Do you think that cervical cancer preventable?**		
Yes	336	57.6
No	247	42.4
By which method can it be prevented?*		
Sexual abstenance	60	10.3
Being faithfull to partner	67	11.5
Using condom	40	6.9
Vaccination	92	15.8
Others	43	7.4
I don’t know	197	33.8

*More than one options are reported by a participant

Regarding knowledge on vulnerability for the Pap smear test, 254 (43.6%) of the respondents pointed out that all women of child bearing age should get the pap smear test but more than a quarter, 216 (37%) of them reported that they had no information which group of women should get the pap smear test for screening cervical cancer (Table 2).

Less than half of women reported that cervical cancer is not a preventable disease. Among women who reported the disease is preventable, approximately half, 288 (49.4%) of the respondents mentioned early detection and treatment are means of preventing cervical cancer. And risk reduction and vaccination were also prevention methods of cervical cancer cited by 116 (19.9%) and 92 (15.8%) of the respondents respectively (Table 2).

### Practice of screening for cervical cancer

Of all the participants, only 58 (9.9%) of them had been screened for the cervical cancer before the survey. All the participants who had been screened are those who had the intention to be screened for the disease. Those who were informed about the services but not yet screened for cervical cancer had mentioned reasons like unavailability of the service nearby 20 (3.4%), unaware of where to get the service 12 (2.1%), financial problem 3 (0.5%), fear of discrimination 2 (0.3%) and other reasons 5(0.9%).

**Fig 1:**
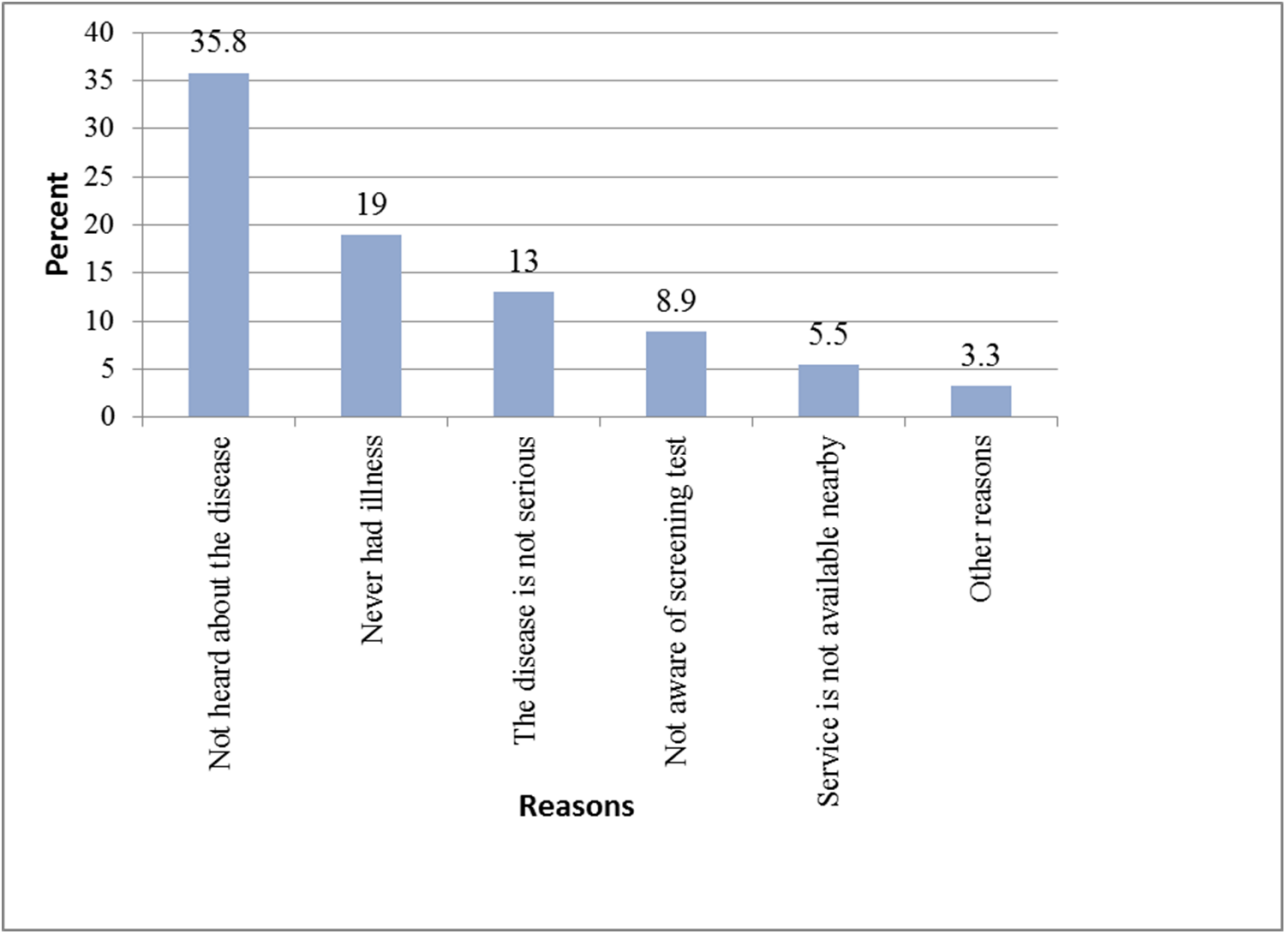
Major reasons for not seeking health for cervical cancer among respondents.

Majority 500 (85.8%) of participants had no intention to be screened for cervical cancer. With regard to the reasons why participants were not seeking health for cervical cancer,majority, 209 (35.8%) of the participant indicated that they had never heard about the disease followed by never had experienced the illness before, 111 (19%) (Fig 1).

Whereas 83 (14.2%) of participants reported that they had the intention to be screened for the disease in any health facility.Among those who had intention to be screened, almost all, 76 (91.6%), of the participants gave a reason that someone recommended the service for them before the survey. The health workers 34 (44.%), neighbors 17 (22.4%), colleagues 10 (13.2%), spouse 8 (10.5%) and relatives 7 (9.2%) were groups who mostly recommended the screening service for the respondents.

### Attitude towards cervical cancer prevention

Based on operational definition stated in method section, 203 (34.8%) of participants had negative attitude while 380 (65.2%) of respondents had positive attitude towards selected proxy variables.

Only 64 (11%) of study participants believed that they needed awareness about cervical cancer as they were of the opinion that they can have the disease at any point in time while 76(13%) of them had no idea. About a quarter of the study participants believed the cervical cancer as a deadly disease to them if they are not screened at the earliest possible time and treated whereas 100(17.2%) of them were neutral. Likewise, 225(38.6%) and 252(43.2%) of the participants perceived that they can have cervical cancer through unsafe sexual practice and all females are at risk of getting the disease respectively. Three hundred eighteen (54.5%) of the respondents opposed the idea to which they couldn’t be cured from the disease once they have had it. Majority, 540(75.4%) of the participants have felt the benefits of regular medical check-up for the disease. Two hundred seventy five (45.4%) of the participants supported the idea that cervical cancer cannot lead them to be infertile if left untreated (Table 3).

**Table 3:** Attitude of respondents about perceived susceptibility and severity, benefit of screening and prevention of cervical cancer in Hossana town, Hadiya Zone, June 2015.

Variables	Level of agreement, number (%)				
	Strongly Agree	Agree	Neutral	Disagree	Strongly Disagree
one need not to be aware of cervical cancer because I cannot have it at any exposure.	62 (10.6)	381(65.4)	76(13.0)	61(10.5)	3(0.5)
Cervical cancer is a deadly disease if not screened and treated.	38(6.5)	105(18.0)	100(17.2)	282(48.4)	58(9.9)
One can have cervical cancer through unsafe sexual practice	15(2.6)	210(36.0)	248(42.5)	104(17.8)	6(1.0)
One cannot be cured from cervical cancer once a diagnosis is made.	38(6.5)	169(29.0)	58(9.9)	288(49.4)	30(5.1)
Cervical cancer cannot lead one to be infertile if left untreated.	5(0.9)	121(20.8)	192(32.9)	252(43.2)	13(2.2)
It is perceived that all female are at risk of having Cervical cancer.	23(3.9)	229(39.3)	145(24.9)	137(23.5)	49(8.4)
One derives great benefit by going to the clinic for regular medical check-up	122(20.9)	318(54.5)	108(18.5)	35(6.0)	0(0)

### Health service related factors

Ninety five (16.3%) of the respondents had ever received information about the cervical cancer from the health workers. Government health facilities, 67(11.5%), privatee health institutions 20 (3.4%) and other sources 8 (1.4%) were the commonest sources of information about the cervical cancer. Only, 85 (14.6%) of the respondents actively sought information related to the cervical cancer from different sources prior to this study. Among participants who sought information about cervical cancer, Television 67 (11.5%,) health professionals 24 (4.1%), Radio 23 (3.9%), internet 21 (3.6%), Magazine 19 (3.3%), and newspaper 8 (1.5) were the sources of information.

### Factors associated with knowledge of and attitude towards cervical cancer

Participants who did not have health seeking behavior were about times 5.45 more likely to have poor knowledge score when compared to those who have health seeking behavior for prevention and control of cervical cancer [AOR: 5.45, 95% CI: (1.18,30.58)]. Similarly, those who had never received information about cervical cancer and its prevention were about 2.63 times more likely to have poor knowledge score when compared to those who had ever received information from any sources [AOR: 2.63,95%CI: (1.78,8.84)]. In the same manner, participants who were not actively seeking health information about cervical cancer were 6.25 times more likely for having poor knowledge score as compared to those who were actively searching health information about cervical cancer [AOR: 6.25, (95%CI: (1.26, 31.06)]. However, other socio demographic factors were not statistically significant with poor knowledge score for cervical cancer prevention and control (Table 4).

**Table 4:** Factors tested for association with cumulative knowledge score about cervical cancer among the respondents in Hossana town Hadiya Zone, June, 2015.

Variable	Variable category	Freq	Crude OR 95%CI	P-value	Adjusted OR 95% CI	P value
Age	≤ 23	135	0.95(0.60,1.53)	0.861		
	24–27	128	0.69(0.43,1.11)	0.123		
	28–34	170	0.75(0.48,1.17)	0.202		
	≥ 35	150	1			
Income	<72	134	2.07(1.25, 3.29)	0.004	0.75(0.31,1.82)	0.531
	72–143	212	1.95 (1.26, 3.03)	0.003	1.26(0.66,2.41)	0.478
	144–215	97	0.95 (0.57, 1.69)	0.959	0.50(0.24,1.07)	0.074
	≥215	140	1		1	
Religion	Orthodox	123	1			
	Protestant	388	0.81 (0.43, 1.52)	0.712		
	Islam	33	0.93 (0.29, 3.04)	0.853		
	Others	39	0.29 (0.12, 0.69)	0.253		
Respondent’s education	No education	74	37.19 (5.03, 274.89)	0.018	2.65(0.84,8.36)	0.096
	Primary (1–8)	172	8.25 (4.03, 16.92)	0.015	1.97(0.78,4.99)	0.152
	Secondary (9–12)	177	4.50 (2.50, 8.10)	0.012	1.05(0.44,2.54)	0.912
	Tertiary (12+)	160	1		1	
Husband’s occupation	Employee	155	1		1	
	Merchant	140	1.36 (0.85,2.18)	0.195	0.69(0.32,1.49)	0.340
	Farmer	21	4.55 (1.67, 12.38)	0.003	1.13(0.33, 3.07)	0.849
	Daily worker	30	2.73 (1.22, 6.08)	0.014	0.61(0.21,1.81)	0.373
	Others	43	3.07 (1.52, 6.18)	0.002	1.48(0.56,3.92)	0.429
Husband’s Education	No education	14	6.11(1.82,20.49)	0.003	2.793(0.592,13.18)	0.194
	Primary (1–8)	98	5.28(3.05,9.15)	0.001	2.614(1.12,6.12)	0.027
	Secondary (9–12)	122	1.88(1.14,3.09)	0.013	1.215(0.55,2.68)	0.629
	Tertiary (12+)	155	1		1	
Parity	0	162	1			
	1	82	1.52(0.89,2.60)	0.122		
	2–4	238	0.98(0.66,1.47)	0.940		
	≥ = 5	101	2.02(0.22,3.35)	0.116		
Health Seeking behaviour for cervical cancer *	Yes	83	1		1	
	No	500	46.78(11.38,192.37)	0.001	5.45(1.18,30.58)	0.031
Ever received information*	Yes	95	1		1	
	No	488	17.48(7.51,40.72)	0.001	2.63(1.78,8.84)	0.018
Active health information seeking*	Yes	85	1		1	
	No	498	31.59 (9.85, 101.33)		6.25(1.26,31.06)	0.025

*significantly associated factors

Among variables entered in bivariate and multivariate logistic regression analysis, only knowledge score was associated with poor attitude with wide confidence interval i.e low precision. Participants who had poor knowledge score were 56.5 more likely to have negative attitude [AOR: 56.51, 95%CI: (23.76, 134.37), P <0.001].

### Factors associated with screening practices for cervical cancer

Despite the fact that precision was very low with wide confidence interval, women who had not ever received information were 45.2 more likely not to be screened for cervical cancer than those who had ever received information about the disease from any source [AOR: 45.24, (95%CI: (11.47,178.54)] (Table 5).

**Table 5:** Factors tested for association with screening practices for cervical cancer among the respondents in Hossana town Hadiya Zone, June, 2015.

Variable	Variable category	Freq	Crude OR 95%CI	P-value	Adjusted OR 95% CI	P value
Age	≤ 23	135	3.30(1.29, 8.50)	0.013	0.58(.08, 4.39)	0.597
	24–27	128	1.64 (0.75, 3.56)	0.214	1.67(0.38, 7.35)	0.497
	28–34	170	1.09(0.57, 2.10)	0.793	2.53(0.69, 9.17)	0.158
	≥ 35	150	1	1	1	
Income (USD)	<72	134	9.03(0.65, 30.79)	0.071		
	72–143	212	1.88 (0.05, 3.53)	0.069		
	144–215	97	1.80(0.82, 3.96)	0.144		
	≥215	140	1			
Religion	Orthodox	123	1			
	Protestant	388	0.87(0.43, 1.77)	0.719		
	Islam	33	0.98(0.26, 3.75)	0.979		
	Others	39	0.66(0.22, 2.06)	0.482		
Respondent’s education	No education	74	22.74(3.06,169.13)	.002	1.35(0.10, 18.75)	0.824
	Primary (1–8)	172	5.64(2.63, 12.11)	.000	0.45(0.08, 2.49)	0.359
	Secondary (9–12)	177	5.20(2.49, 10.84)	.000	1.49(0.36, 6.18)	0.587
	Tertiary (12+)	160	1		1	
Husband’s occupation	Employee	155	1		1	
	Merchant	140	5.52(2.91, 10.45)	.000	3.13 (0.93, 10.55)	.065
	Farmer	21	8.62(2.55, 29.21)	.001	0.84 (0.03,25.20)	.922
	Daily worker	30	31.96(4.28, 24.42)	.001	3.63 (0.16,83.35)	.420
	Others	43	6.21(1.82, 21.24)	.004	0.94 (0.16, 5.62)	.941
Ever received information^*^	Yes	95	1		1	
	No	488	121.90 (46.23, 321.45)	0.000	45.24(11.47,178.54)	.001
Active health information seeking^*^	Yes	85	1		1	
	No	498	32.97(16.92, 64.23)	.000	6.96(2.14, 22.64)	.001
Knowledge score^*^	Good	313	1		1	
	Poor	270	59.89(8.23, 435.75)	.000	11.124(1.01,122.26)	.049

*significantly associated factors

## Discussion

Knowledge, attitude and practice of the community about any disease including cervical cancer and its factors offer crucial opportunity for comprehensive prevention and control strategies of the disease. Therefore, this study addressed knowledge, attitude and practice as an entry point for the prevention and control of cervical cancer and its associated factors among women of child bearing age.

The findings of this study showed that less than half (46.3%) of participating women had poor level of comprehensive knowledge score from the composite score regarding vulnerable groups, risk factors, signs and symptoms and methods of prevention of cervical cancer. This finding is lower than a similar the study done in Addis Ababa among female ART attendants [16]. This could most probably be due to the participants’ exposure to information through health professionals while they undergo regular follow up for ART services. Although there is a slight difference in knowledge score construction, the level of knowledge in this study is also lower than the study in North West of Ethiopia [15]. This difference might be due to the practice of community health education in the northern part of the country that takes an advantage of the University of Gonder which is a pioneer in practicing a community based education in the area. The finding of this study is also lower than that of African cervical cancer studies of Osun and Lagos in Nigeria [17–19].

Less than half, 254 (43.6%) of the respondents believed that all women are at risk of getting cervical cancer while 216 (37.0%) of them did not know which women are at risk of getting the disease. This finding showed that participants had much lower awareness level about risk groups when compared to findings of other studies done in Ethiopia [18, 20] and other countries [17, 21, 22]. This could be attributed to low attention given to media promotion, variations in health information provision about cervical cancer and its exposure. In addition,differences in socio-cultural conditions, health education at healthcare facilities and other behavioral change interventions regarding the cervical cancer prevention and control program of Ethiopia.

As observed from the study, 223 (38.3%) of the participants had no idea what factors increased the chances of getting cervical cancer. Majority of the participants were not able to cite the risk factors for cervical cancer. Only about nine in hundred indicated infection with human papillomavirus as a risk factor, but more than a quarter of participants cited at least one risk factor related to having multiple sexual partnership. These misconceptions were more reflected in this study than the study done in North West of Ethiopia [15]. This even could be as a result of selection bias of the health information while they attend any health care services delivery about other sexually transmitted infections. These findings are far more difficult to compare with the findings of other studies due to differences in rating multiple responses among studies. The discrepancy between knowledge of women about citing early detection and treatment and low health seeking behavior for screening could be explained by inability to bring a behavioral change. On the other hand, women might believe that regular checkups for other health problems could work for cervical cancer too without clearly knowing correct methods of prevention and control of cervical cancer.

As indicated from this study, major risk factors reported include multiple sexual partners. Although having sex with one sexual partner is sufficient to acquire infection with HPV, having multiple partner is an important risk factors [23]. This finding is much lower than a similar study done in Africa [17,24]. While infection with human papilloma virus was the major risk factor for occurrence of cervical cancer, only 52(8.9%) of participants reported so. This finding is lower than the finding in Northern region of Ethiopia [15]. But nearly consistent with the finding in Gabon in Africa, 8% [25].

As observed from the study, 336(57.6%) of participants reported that cervical cancer is a preventable disease. Our finding lower than similar study in semi urban part of India; 37 (12.2%) [26]. Our finding is very much lower than the finding in Northern part of Ethiopia [15]. Yet, majority have no information on how the disease can be prevented. Only 40 (6.9%) of participants mentioned condom as a method of prevention while it is effective method of primary prevention. The disease is preventable given behavioral interventions focusing on individual, societal and policy changes [12] as well as biological interventions like vaccination [27] are well implemented. Similarly, very few of participants reported that vaccination could be a preventive method. Despite vaccination, not being implemented in Ethiopia, the awareness and knowledge of participants would be indispensible so that the future implementation will be utilized.

Two hundred three (34.8%) of participants had negative attitude towards selected proxy variables. Our finding is lower than other findings in Ethiopia [15], although the score we used for attitude is different.

Only 58 (9.9%) of respondents had been screened for the cervical cancer before the survey. This finding is consistent with hospital based finding in Nigeria among women of reproductive age [28]. This study has showed that only 14.2% of participants reported that they had the intention to be screened for cervical cancer in any health facility. This finding is about four folds less than the study done amongst urban women in Malaysia [29]. This finding indicates that the behavioral intervention for prevention and control of the disease had got low attention as women tried to mention their reasons for not having an intention to be screened so that the level of awareness about screening behavior among women is low. Although there are limitations of quantitative findings, the results of this study had been supported by qualitative findings done in south west of Ethiopia [30]. This finding is also consistent with the study done in Africa [13, 17]. Among women who had intention to be screened, more than half of them had been screened before the survey while the rest did not mainly due to service inaccessibility, lack of information about where to get the service, and financial problems. These reasons were factors as indicated by the studies done in the country as well as outside the country [29, 30, 31, 32].

With the contextual categorization of health service related factors, less than a quarter of the respondents had mentioned that they had ever received information about cervical cancer screening from health professionals. Women who have not had an intention to be screened reported reasons like unavailability of the service, unaware of where to get the services and financial constraints. Findings of this study are supported by other studies done in low resource settings [4, 17, 15]. Health service factors such as poor availability, poor accessibility, and poor quality of care provided attributed to women’s lack of information, and to cultural and behavioral barriers [33].

Findings from the multivariate logistic regression in the study showed that not having health seeking behavior for cervical cancer, had never received information about cervical cancer and its prevention and not actively seeking health information about cervical cancer were significantly associated factors with poor knowledge. This finding was congruent with one systematic review finding [34] and finding in Northern part of Ethipia [35]. Those women who had poor knowledge score were more likey to have negative attitude towards proxy factors of cervical cancer. Due to of the scoring difference in finding, it is difficult to compare with other findings. However, knowledge and attitude are the two most interlinked domains for prevention and control of cervical cancer [30]. Women who had never received information about the disease from any source were more likely not to undergo the screening. This finding is supported by the findings in Nigeria [33] and systematic review [30]. Poor knowledge score was not associated with cervical screenening in this study. This finding is contradictory with the finding in Northeast Ethiopia which showed poor knowledge regarding cervical cancer screening [36]. This could be due to the fact that provision of information about cervical cancer is rare which could’t bring any diffenreces among the participants. These results have been supported by findings of other researches about women’s health seeking behavior for cervical cancer sreeening [15]. However other socio-demographic and health service related factors were not found to be statistically associated with any of the three domains. This finding is inconsistent with the study done in North West Ethiopia and findings outside the country [4]. This difference could be explained by the descriptive nature of the study suggesting its inability to detect the real significant difference between factors like very low awareness about the disease and not seeking health for cervical cancer.

## Conclusion

This study highlighted the importance of awareness creation, increasing knowledge, promoting active searching for health information and experiences of receiving information from any information sources regarding cervical cancer. The findings also strongly indicated that different health information dissemination strategies may be required for women to increase knowledge about and attitude towards prevention and control of cervical cancer. We recommend greater attention to the adaptation of comprehensive prevention packages for cervical cancer by integrating with other reproductive health services like antenatal care services, health information education and communication at all level of health care delivery system in the study area. Further more, it’s important to use proven approaches in implementation of health extension programs like health development army to encourage screening behavior of women by all health care providers and other health sector stakeholders. Finally, research is needed to fully understand the issues relevant to women who had no screening behavior for prevention and control of cervical cancer.

## Limitation of the study

The study did not address the role of the partner the of the study participants. Those participants who scored for screening is very small so that the model of multivariate analysis may not be stable.

## Supporting information

S1 Dataset Data set for knowledge,attitude and practice for cervical cancer, Hossana town Ethiopia. (SAV)

## Abbreviations

HPV: human papilloma virus
IARC: Information Agency for Research on Cance
SVA: Single Visit Approach
VIA: Visual inspection with Acetic acid
IEC/BCC: Information Education and communication/Behavioural change communication
GAVI: global alliance for vaccination and immunization
SNNPR: south nations, nationalities and people regional state
KM: kilometer
ETB: Ethiopian Birr
TV: Television
HIV: human immune deficiency virus
AIDS: acquired immune deficiency syndrome
ART: Antiretroviral therapy
